# Function of the p38MAPK-HSP27 Pathway in Rat Lung Injury Induced by Acute Ischemic Kidney Injury

**DOI:** 10.1155/2013/981235

**Published:** 2013-03-27

**Authors:** Tao Ma, Xiao Wei Liu, Zhi Liu

**Affiliations:** Department of Emergency Medicine, The First Hospital of China Medical University, No. 155 Nanjing North Street, Heping District, Shenyang City, Liaoning Province 110001, China

## Abstract

This study aims to observe the changes and the function of p38MAPK-HSP27 signaling pathways in acute lung injury (ALI) induced by acute ischemic kidney injury in rats. Wistar rats were randomly divided into Group A (control group), Group B (acute kidney injury group), and Group C (acute kidney injury +SB203580). The concentration of protein in BALF, neutrophil counts, PI, W/D; the concentration of TNF-**α**, IL-6, and IL-1**β** in plasma and BALF; and the concentrations of MDA and NO in the lung tissue started to increase 2 h after the experiment in Group B, which showed a significant difference compared with those in Groups A and C. The expressions of p-p38MAPK and p-HSP27 in the lung tissue began to increase 2 h after the experiment in Group B, which was different from those in Groups A and C. A significant increase was observed in the F-actin expression in Group B than that in Group A. In Group B, the correlation of cytokine TNF-**α**, IL-6, and p-p38MAPK in BALF was positive. Acute kidney injury (AKI) induced by bilateral renal arteriovenous clamp closure could activate p38MAPK-HSP27 signaling pathways and induce lung injury, which blocks the p38MAPK-HSP27 signal pathway to reduce the risk of lung injury.

## 1. Introduction

AKI, one of the common but complicated clinical issues, is experienced in 30% of critical patients. About 5%-6% of the patients in the intensive care unit can suffer from AKI and need renal replacement [1]. Despite the continuous improvement of hemodialysis and advanced therapies, the mortality of AKI remains as high as 40%–60%. In fact, the usual cause of death is not the renal failure itself. Acute lung injury (ALI), either cardiogenic or noncardiogenic, can be the cause of the high mortality of AKI [2]. Studies have shown that AKI is usually developed with ALI, especially with acute respiratory distress syndrome, in which the mortality is nearly 80% [3–5]. A high mortality of AKI is significantly correlated with injuries of remote organs [6–8]. However, little information is available on the mechanisms of the induction of ALI after the onset of AKI.

ALI is generally aggravated by nephrogenic pulmonary edema, that is, increased capillary transmural hydrostatic pressure (similar to the increased volume load in heart failure) because of water-sodium retention caused by acute renal failure. Based on recent studies on animals, however, the changes in pulmonary vascular permeability vary at the very onset of AKI [9, 10], which could cause lung injury. These findings challenge the classical recognition of lung injury, as secondary to kidney injury. Recent studies have found similar variations in inflammatory factors, such as CD14, serum amyloid protein A, lipocalcin, and IL-1R. In the lung and in the kidney after AKI, a positive correlation exists between the levels of inflammatory factors and kidney injury duration [11–14]. This correlation implies that inflammatory factors and cytokines have important function in lung injury, as secondary to kidney injury. However, the mechanism of pulmonary vascular permeability changes under the influence of cytokines after acute renal injury, and the induction of lung injury remains unknown.

As shown by previous studies, the signal pathway of p38 MAPK-HSP27 regulates the assembly/disassembly of actin, reconstructs the cytoskeleton, and changes the pulmonary vascular permeability [15]. The present study aims to observe the trends in the variation of IL-6, TNF-*α*, and the p38MAPK-HSP27 pathway in ALI induced by AKI to explore the pathogenesis and provide a laboratory and theoretical basis for early-stage diagnosis and therapy in ALI, as secondary to AKI.

## 2. Material and Methods

### 2.1. Establishment of the Animal Model and Randomization

About 90 healthy male Wistar rats weighing 300 g–320 g from the animal center of the China Medical University with postintraperitoneal anesthesia (5% chloral hydrate) underwent tracheotomy and catheterization in carotid arterial and jugular vein. The respiration, heart rate, diastolic/systolic pressure, mean arterial pressure, and central venous pressure of the rats were monitored. The rats that were in stable condition for 30 min were randomly divided into three groups with 30 rats in each group. In Group A (control group), bilateral arteriovenous were separated, and bilateral renal arteriovenous were not ligated. Intraperitoneal injection was performed with 2 mL of a saline solution. In Group B (AKI group), bilateral arteriovenous were separated and bilateral renal arteriovenous were ligated. Intraperitoneal injection was then performed with 2 mL of a saline solution. In Group C (AKI +SB203580), bilateral arteriovenous were separated, and bilateral renal arteriovenous were ligated. Intraperitoneal injection was then performed with 2 mL of SB203580 (the inhibitors of p38MAPK, namely, pyridine imidazole medicament derivatives at 2 mg/kg). This study was carried out in strict accordance with the recommendations in the Guide for the Care and Use of Laboratory Animals of the National Institutes of Health. The animal use protocol has been reviewed and approved by the Institutional Animal Care and Use Committee (IACUC) of the First Hospital of China Medical University.

### 2.2. Sample Collection and Test

After the establishment of the model, six animals in each group were killed at 0, 2, 4, 6, and 8 h. The cytokine in venous blood was tested. Lungs exposed after thoracotomy were observed for gross changes. After the right hilum was ligated, the upper-right lung was taken, fixed with 4% paraformaldehyde, embedded in paraffin, sliced, HE-stained, and observed for pathological changes by using a microscope. The lower-right lung was then observed. The blood was eliminated using a filter, weighed, and heated in an oven (80°C for 72 h) to obtain a stable weight. The blood was then weighed to determine its dry weight, and the (W/D) weight ratio was then calculated. Bronchoalveolar lavage was performed on the left lung in triplicate with 3 mL of a phosphate buffer solution per test. The collected BALFs in the three tests were mixed together and cryopreserved for cytokine analysis. Coomassie brilliant blue staining was performed to identify the protein in BALF. The rats received intravenous injections of human serum albumin (HSA) an hour before extermination. The HSP levels in the plasma and in BALF were determined by enzyme-linked immunosorbent assay (ELISA). PI was calculated as the BALF-to-plasma ratio of the HSA concentration. The sediments of BALF were used to determine the neutrophil count.

### 2.3. Hematoxylin and Eosin (HE) Staining

Lung tissue was normally fixed, dehydrated, embedded, and sliced to obtain hematoxylin- and eosin- (HE) stained slices. Multiple high-power fields were randomly selected for each HE-stained slice to compare the variations in lung tissues and cells.

### 2.4. Test for IL-1*β*, IL-6, IL-10, and TNF-*α*

Double antibody sandwich ELISA was used to determine the concentrations of IL-1*β*, IL-6, IL-10, and TNF-*α* in the plasma and in BALF, according to the instructions in the assay kit (Shanghai Senxiong Technology Company, Shanghai, China).

### 2.5. MDA and NO Tests

The middle lobe of the right lung that was taken after thoracotomy was immediately cryopreserved in liquid nitrogen and made into a homogenate. Thiobarbituric acid and nitrate reductase methods were used to determine the MDA and NO contents, respectively, according to the instruction in the assay kit (Beyotime).

### 2.6. Western Blot

From the obtained homogenate in Section 1.5, the protein was extracted and its concentration was measured. The homogenate was kept under −80°C. A 100 *μ*g protein sample was used for electrophoresis in 12% sodium dodecyl sulfate polyacrylamide gel electrophoresis, which ended when the targeted proteins reached the bottom of the gel. The targeted proteins were transferred on a polyvinylidene fluoride membrane under 4°C at a permanent voltage of 120 V and concealed for 1 h with 5% skimmed milk powder at room temperature. A 1 : 1000 diluted p-p38MAPK (SANTA), a primary antibody of p-HSP27 (SANTA), an antibody of F-actin (Abcam), an antibody of G-actin (SIGMA), and a 1 : 500 diluted primary antibody of glyceraldehyde 3-phosphate dehydrogenase (internal reference) were added. The proteins were incubated overnight at 4°C with the addition of a secondary antibody labeled with horseradish peroxidase (1 : 1000). The solution was then incubated at room temperature for 1 h. The sample was observed for luminescence and was photographed using the gel imaging system. The results were analyzed using Quantity One, and the optical density was scanned.

### 2.7. Statistics

SPSS 16.0 was used in statistical calculations. Count data were expressed in $\overset{-}{x}\pm s$, and *t*-test was performed for intergroup comparison. Pearson correlation analysis was performed for correlation analysis between two groups. *P* < 0.05 was considered as significant difference.

## 3. Results

### 3.1. Variation of Protein Level and Neutrophil Counts in BALF

The degree of ALI was evaluated by the protein content and neutrophilic count in BALF. The protein content and neutrophilic count had no significant variations at each time point after the experiment in Group A. Both values in BALF began to increase 2 h after the experiment in Group B and maintained a gradual increasing trend with significant difference compared with Group A. Compared with Group B, both values in BALF were significantly decreased in Group C, which was treated with SB203580. The difference was statistically significant (*P* < 0.05) (Table 1).

### 3.2. PI Variation and (W/D) Lung Weight Ratios

The permeability of pulmonary vascular endothelial cells or alveolar epithelial cells was evaluated via PI and W/D. PI and W/D had no significant variations at each time point after the experiment in Group A. Both values began to increase 2 h after the experiment in Group B and maintained a gradually increasing trend with significant difference compared with Group A. When compared with group B, both values significantly decreased in Group C. The difference was statistically significant (*P* < 0.05) (Table 2).

### 3.3. Concentration of TNF-*α*, IL-1*β*, IL-6, and IL-10 in Plasma and BALF

The concentrations of TNF-*α*, IL-1*β*, IL-6, and IL-10 in plasma and BALF reflect the inflammation level of the rats. The concentrations of TNF-*α*, IL-1*β*, IL–6, and IL-10 in plasma and BALF were measured by ELISA, wherein no significant variation was observed at each time point after the experiment in Group A. The concentrations of TNF-*α*, IL-1*β*, and IL-6 in plasma and BALF began to increase 2 h after the experiment in Group B and maintained a gradually increasing trend. This result is significantly different with Group A. Compared with Group B, the concentrations of TNF-*α*, IL-1*β*, and IL-6 in plasma and BALF significantly decreased in Group C. The difference was statistically significant. However, the anti-inflammation (IL-10) 2 h after the start of the experiment had a gradually increasing trend, and then a gradual decline was observed 4 h after the peak was reached. Compared with Group B, no significant difference was observed in Group C. No statistical significance was observed in the difference (*P* > 0.05) (Table 3).

### 3.4. Concentration of MDA and NO in Lung Tissue

MDA and NO reflect the level of lipid peroxidation. The MDA and NO contents in the rat lung tissue were measured by thiobarbituric acid and nitrate reductase methods, respectively, to evaluate the level of lipid peroxidation in the rat lung tissue. Both values had no significant variation at each time point after the experiment in Group A. Both values began to increase 2 h after the experiment in Group B and maintained a gradual increasing trend with significant difference compared with Group A. Compared with Group B, both values significantly decreased in Group C. The difference was significantly significant (*P* < 0.05) (Table 4).

### 3.5. Pathology of Acute Kidney Injury (AKI) and Lung Injury Induced by SB203580

**Figure 1:**
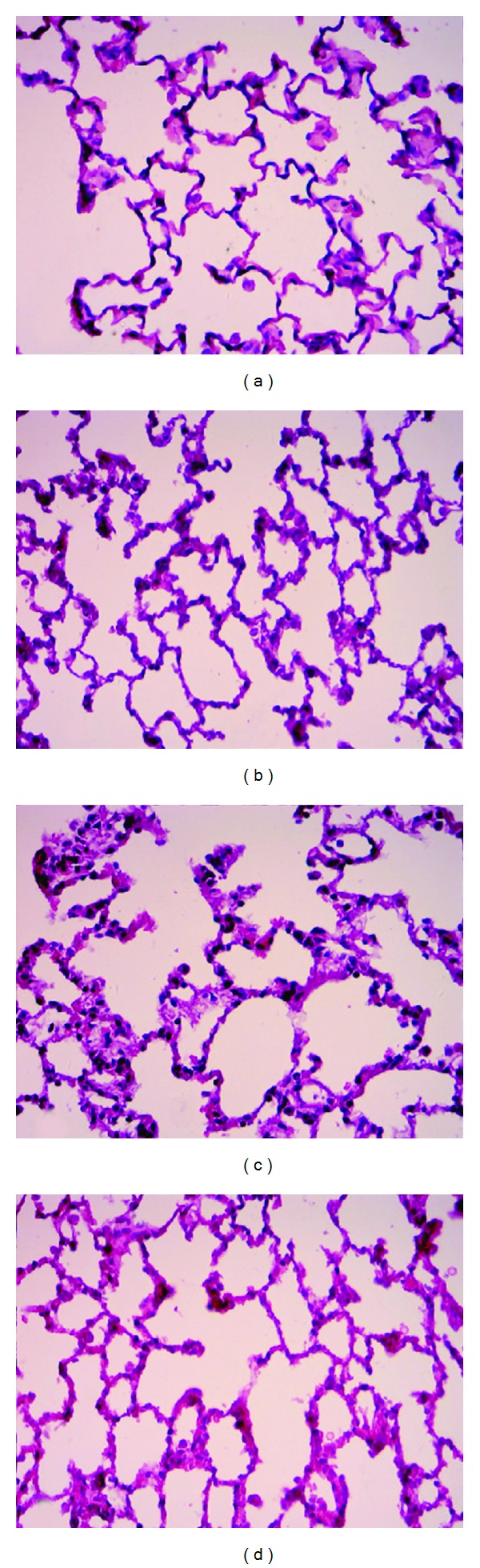
Morphology of the lung tissues of different groups after HE staining. (a) and (b) The lung tissue of the control group (Group A) at 0 and 8 h, respectively, in which the alveolar structures are integral without effusion from inside the alveoli or pulmonary interstitial edema; (c) the lung tissue of the acute lung injury group, in which swollen alveolar epithelia, thickened alveolar walls, telangiectasia and capillary hyperemia, and pulmonary interstitial edema, noticeably increased effusion of inflammatory cells, red blood cells, and proteins from inside the alveoli, and disordered alveolar structures are observed, showing pathological changes of acute lung injury; and (d) the lung tissue of Group C after the administration of the p38MAPK inhibitor SB203580, in which effusion of inflammatory cells, red blood cells, and proteins from inside the alveoli is observed, but the interstitial and alveolar edemata are milder compared with Group B.

The changes in the morphology were observed by HE staining to evaluate the effect of acute renal injury and SB203580, which were the inhibitors of P38 on pulmonary morphology. The alveolar structure appeared intact without exudates in the alveoli or pulmonary interstitial edema in the normal control group at 0 and 8 h. In the kidney injury group (Group B), swollen epithelium was found with thickened alveoli wall, as well as telangiectasia, capillary hyperemia, evident edema in the stroma and cavity of alveoli, and inflammatory cells in the alveoli cavity. The exudates of red blood cells and protein increased because of the infiltration of numerous inflammatory cells. In other areas, injury in the small air tracts was observed with disorder in the alveoli structure, which indicates the pathological changes in ALI. In Group C, where the inhibitor of p38MAPK (SB203580) was used, exudates of inflammatory cells, red blood cells, and protein were observed in the alveoli. However, the edema in the stroma or cavity of alveoli was mild (Figure 1).

### 3.6. Expressions of p-p38MAPK and p-HSP27

**Figure 2:**
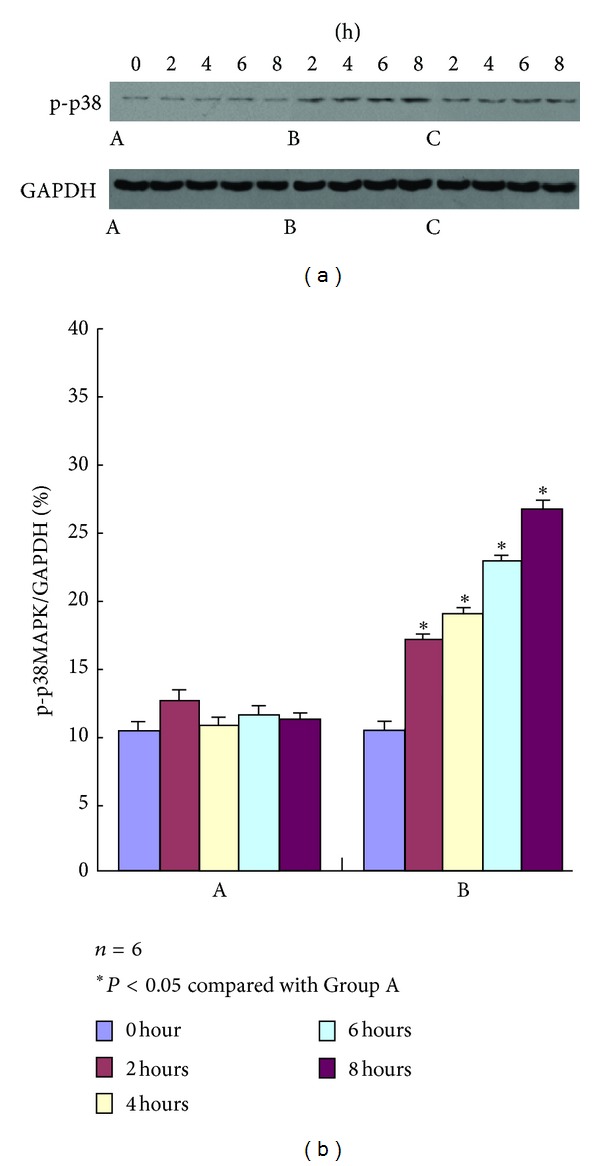
p-p38MAPK expression in Groups A, B, and C at different time points using Western blot analysis. The strip concentration of p-p38MAPK in Group B begins to rise from 2 h after test, whereas that in Group A does not show any significant difference; compared with Group B, p-p38MAPK expression in Group C decreases at different time points after the administration of the p38MARPK inhibitor SB203580.

**Figure 3:**
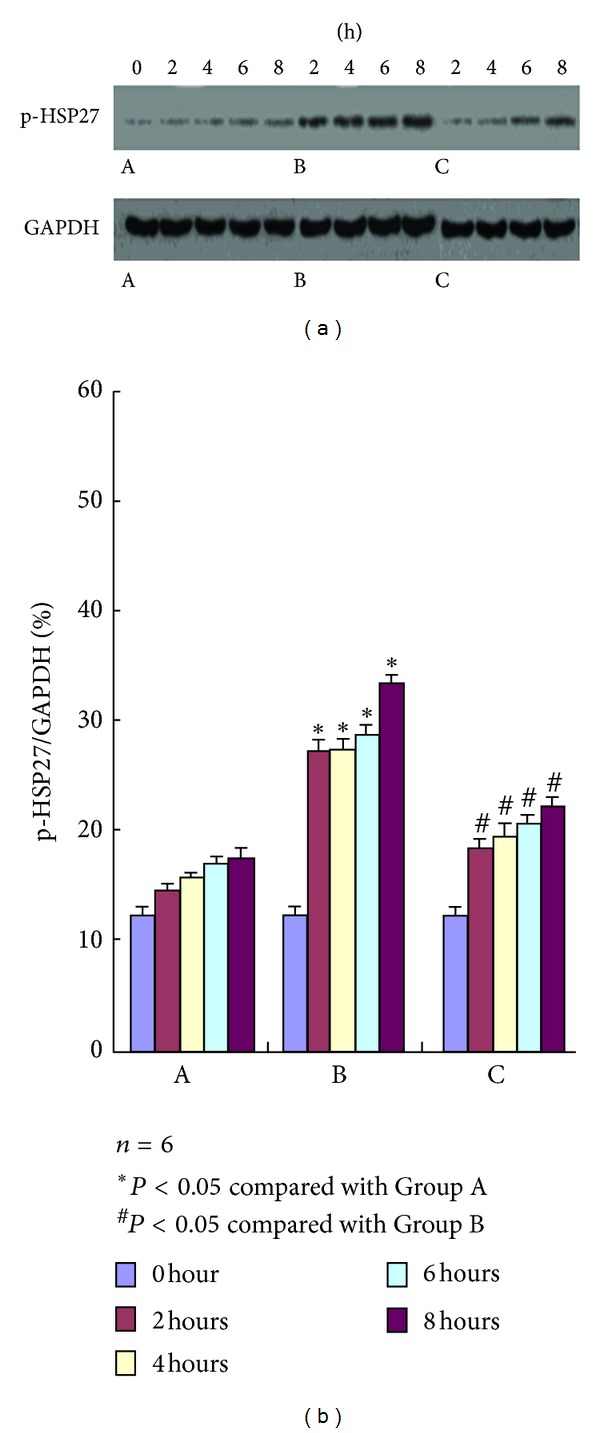
p-HSP27 expression in Groups A, B, and C at different time points using Western blot analysis. The strip concentration of p-HSP27 in Group B begins to rise from 2 h after test, whereas that in Group A does not show any significant difference; compared with Group B, p-HSP27 expression in Group C decreases at different time points after the administration of the p38MARPK inhibitor SB203580.

The concentrations of p-p38MAPK and p-HSP27 were measured by Western blot to evaluate the effect of AKI on the p38 MAPK-HSP27 signal pathway. As shown in Figures 2 and 3, the concentration of the band in the Western blot had no significant variations at each time point after the experiment in Group A. The concentration began to increase 2 h after the experiment in Group B and maintained a gradually increasing trend with significant difference compared with Group A. This result showed that the expressions of p-p38MAPK and p-HSP27 also increased. Compared with Group B, the concentration of the band in the Western blot significantly decreased in Group C. The difference was statistically significant (*P* < 0.05). 

### 3.7. Variation in the Ratios of F-Actin to G-Actin

**Figure 4:**
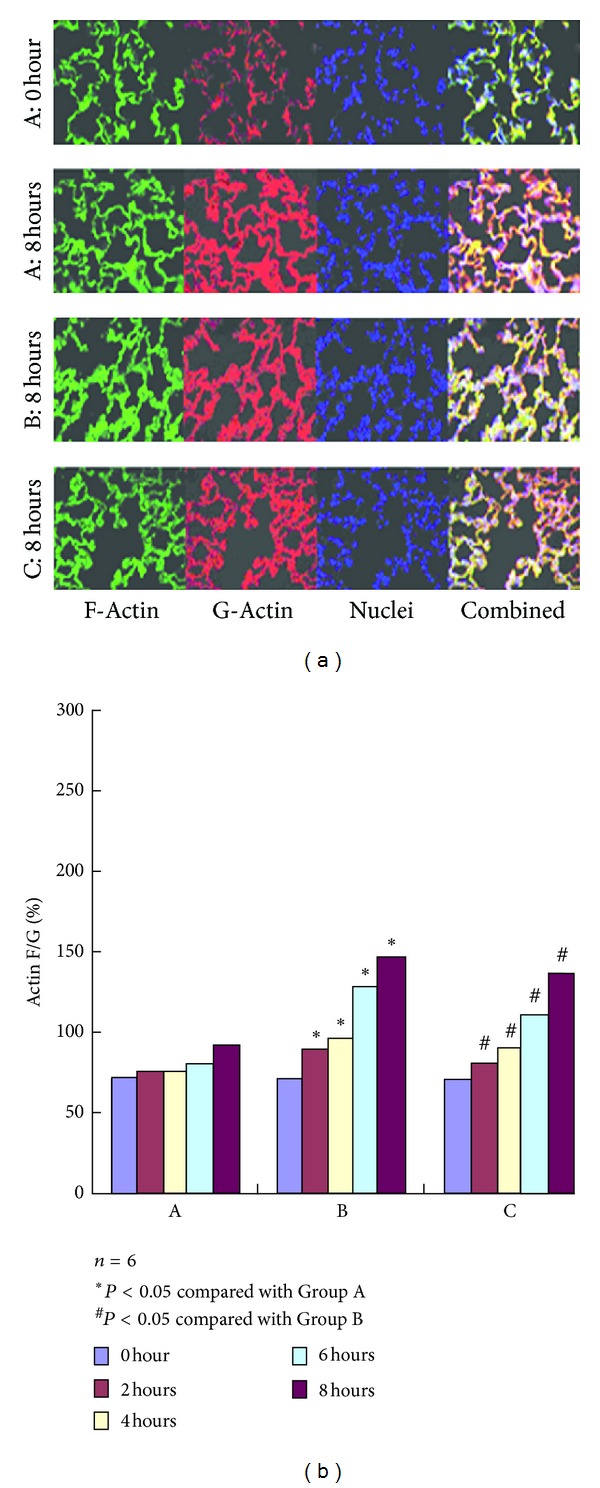
F-actin and G-actin expression in Groups A, B, and C at different time points using Western blot analysis. The concentration of Western blot of banding of F-Actin and G-Actin had no significant variation at each time point after the experiment in Group A. The concentration of Western blot of banding of F-Actin begin to increase at 2 hours after the experiment in Group B, appeared to maintain a gradually increasing trend, but the concentration of Western blot of banding of G-Actin began to decrease, which is a significant difference compared with Group A. Compared with Group B, the concentration of Western blot of banding of F-Actin was significantly decreased, and that of G-actin were significantly increased in group C which was treated by SB203580,  *P* < 0.05. There is statistical significance. As shown in (b) the ratios of F-Actin to G-Actin has no significant variation at each time point after the experiment in Group A. The ratios of F-Actin to G-Actin began to increase at 2 hours after the experiment in Group B and appeared to maintain a gradually increasing trend, significant difference compared with Group A. Compared with Group B, the ratios of F-Actin to G-Actin were significantly decreased in Group C which was treated by SB203580, *P* < 0.05. There is statistical significance.

The concentrations of F-actin and G-actin were measured by Western blot. The ratios of F- to G-actin were calculated to evaluate indirectly the changes in the endothelial cytoskeleton after AKI. As shown in Figure 4(a), the concentrations of the band in the Western blot of F- and G-actin had no significant variations at each time point after the experiment in Group A. The concentration in F-actin began to increase 2 h after the experiment in Group B and maintained a gradually increasing trend. However, the concentration of G-actin began to decrease with significant differences compared with Group A. Compared with Group B, the concentration of F-actin significantly decreased, whereas that of G-actin significantly increased in Group C. The difference was statistically significant (*P* < 0.05), as shown in Figure 4(b).

The ratios of F- to G-actin had no significant variations at each time point after the experiment in Group A. The ratios began to increase 2 h after the experiment in Group B, and maintained a gradually increasing trend with significant difference compared with Group A. Compared with Group B, the ratios significantly decreased in Group C. The difference was statistically significant (*P* < 0.05).

### 3.8. Correlation Analysis on p-p38MAPK, TNF-*α*, and IL-6

**Figure 5:**
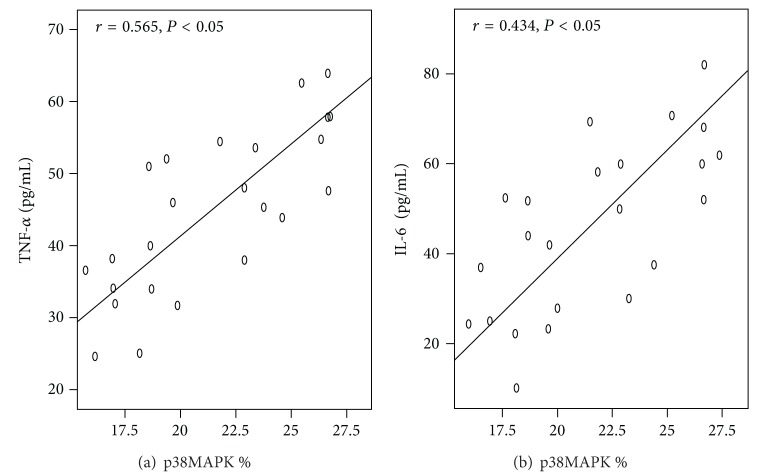
Analyses of the correlations of TNF-*α* and IL-6 with p-p38MAPK in the acute lung injury group. Both TNF-*α* and IL-6 are positively correlated with p-p38MAPK with the correlation coefficients of 0.565 and 0.434, respectively (*P* < 0.05).

Figure 5 shows a positive correlation between p-p38MAPK and TNF-*α* (or IL-6), with correlation coefficients of 0.565 and 0.434, respectively.

## 4. Discussion

This research showed that the protein level and neutrophil counts in BALF significantly increased 2 h after AKI with higher (W/D) lung weight ratios and PI. Pathological changes in ALI, which include swollen alveoli epithelium, expanded alveoli wall, telangiectasia, capillary hyperemia, evident edema in the stroma and cavity of alveoli, and inflammatory cells in alveoli, significantly increased the exudates of red blood cells and protein, the infiltration of numerous inflammatory cells, the injury of small air tracts in several areas, and the disorderly arrangement of alveoli. Previous studies have proven that ALI occurred at the early stage of AKI, which was characterized by the infiltration of inflammatory cells, the hyperemia in alveoli cavity, and the changes in the alveoli structure [16, 17]. However, in this study, lung injury was observed as early as 2 h after AKI. The lung is the most vulnerable organ to injury because it contains many blood vessels.

TNF-*α*, a vital cytokine in mediating ALI, induces the activation of pulmonary endothelial cells, the migration of white blood cells, the degranulation of granulocyte, and capillary leakage. The accumulated edema further obstructs the influx and oxygen exchange of alveoli, thereby causing ARDS. Furthermore, TNF-*α* can interact with multiple cytokines to produce extensive effects. The production of IL-6 increases with the stimulus of LPS, IL-1*β*, and TNF-*α*. In acute injuries and diseases such as burns, surgeries, and sepsis, the contents of TNF-*α*, IL-1*β*, and IL-6 significantly increase. Clinical studies [1, 18] revealed that the levels of TNF-*α*, IL-1*β*, and IL-6 in BALF and plasma of patients with ALI/ARDS increased and were correlated with the pathogenesis of multiple organ failure. In the present study, the increase in TNF-*α*, IL-1*β*, and IL-6 in the plasma and BALF of rats after AKI is significant. This outcome can be explained by the results of the systematic inflammatory reaction because of surgery, accumulated toxic metabolites after AKI, and the decrease in speed in the elimination and inactivation of the inflammatory mediators through kidneys, wherein the TNF-*α*, IL-1*β*, and IL-6 contents in the rat circulation significantly increase.

Anti-inflammatory cytokine (IL-10) content also increased 2 h after AKI, wherein the peak was reached within 4 h. IL-10 content gradually declined after the peak was reached. The balance between inflammation and anti-inflammation was affected, which caused an inflammation imbalance. In addition, numerous inflammatory mediators in the circulation can be deposited in the lungs because of the massive vascular system in the lungs; therefore, the pulmonary levels of TNF-*α*, IL-1*β*, and IL-6 increase, and ALI is induced. MDA and NO are the product of lipid peroxidation, and they reflect the level of lipid peroxidation in the system. The concentrations of MDA and NO in the lung tissue of AKI in rats increase. The increased inflammation factor that activated oxidative stress also caused the occurrence of lipid peroxidation. This phenomenon increased the concentrations of MDA and NO, which causes the occurrence of ALI.

Another important feature of ALI is the undermined integrity and function of the pulmonary endothelial-epithelial barrier. The three-dimensional structure is maintained by a cytoskeleton, which is the vital structure that sustains the normal shape and function of cells. Microfilament is one of the vital cytoskeletons that consists of F-actin and actin-binding proteins, wherein the assembly/disassembly of actins involves multiple cellular activities [19]. As one of the important actin-binding proteins, HSP27 has a vital function in regulating the reconstruction of actin in the cytoskeleton. Currently, nonphosphorylated HSP27 functions as cap-like proteins that are bound to the anode of F-actin [20] to inhibit the assembly of actins. Phosphorylated HSP27 dissociates from the anode of F-actin, which exposes the anode. The anode of the free F-actin can combine with G-actin to initiate the assembly of G-actin, which results in its prolongation. Therefore, the cytoskeleton of the actin is reconstructed and the cellular gap is expanded, which undermine the pulmonary endothelial-epithelial barrier. Therefore, acute injury occurs. In this study, the expression levels of phosphorylated HSP27, F/G-actin ratio, BALF protein concentration, and pulmonary W/D increase correspondingly, which increases the degree of damage in the lungs. These findings support the capability of phosphorylated HSP27 in regulating the assembly of actin and in improving the prolongation of F-actin, which can induce the alteration of intercell connection and aggravate the damage in the integrity and function of the pulmonary endothelial-epithelial barrier.

In this study, p-p38MAPK correlated positively with p-HSP27 expression levels. The expression levels decreased when the inhibitor of p38MAPK (SB203580) was used, thereby proving that p38MAPK regulates the expression of p-HSP27. MAPK is a vital cellular signal transduction system that regulates cellular proliferation, differentiation, apoptosis, and gene expression. p38MAPK is a member of the MAPK family. Based on previous studies, p38MAPK can be activated by UV, proinflammatory cytokines (TNF-*α*, IL-1*β*, and IL-6), hypoxia, hypertonic environment, inhibitor of protein synthesis, ischemia, reperfusion, and lipopolysaccharide [21]. The phosphorylated 38MAPK can regulate downstream MK2 kinase and HSP27, phosphorylate the serine residues at the 15, 78, and 82 sites of HSP27, and induce the dissociation of HSP27 from the anode of F-actin, thereby changing the cytoskeleton [22–24].

Typical ALI is caused by the induction of AKI and bilateral occlusion of renal arteries and veins. The effects include increased protein content in BALF, increased lung W/D and PI with infiltration of inflammatory cells in the alveoli, increased MDA and NO contents, increased inflammatory mediators (TNF-*α*, IL-1*β*, and IL-6), and damaged alveoli structure. Meanwhile, the contents of p-p38MAPK, p-HSP27, and F-actin in the system gradually increased. The inhibitor of p38MAPK (SB203580) decreased the expression of p-HSP27, slowed down the increase of F-actin content, and lowered the degree of damage in the lungs. These findings can be attributed to the activation of p38MAPK by oxidative stress, inflammatory mediators, hypertonic environment, mechanical stress in the nucleus, induced regeneration and reconstruction of F-actin by regulating the activity of HSP27, and changes in intercellular connection. These effects further affected the pulmonary vascular permeability. When the p38MAPK inhibitor (SB203580) was injected into the abdominal cavity of the rat, as presented in the experiment, the downstream MK2 kinase could not be activated, which could not phosphorylate the serine residues at the 15, 78, and 82 sites of HSP27 that binds with the anode of F-actin. Therefore, the degree of lung injury was lowered, which implies that the p38MAPK-HSP27 signal pathway has a vital function in ALI, as induced by AKI.

## Figures and Tables

**Table 1:** Change of protein content, neutrophil counts in rat's Bronchoalveolar lavage (BALF) ($\bar{x}\pm s$, *n* = 6).

Time (h)	Protein content of BALF (g/L)			Neutrophil counts (×10^6^/mL)		
	A	B	C	A	B	C
0	0.165 ± 0.032	0.165 ± 0.032	0.165 ± 0.032	6.25 ± 0.24	6.25 ± 0.24	6.25 ± 0.24
2	0.173 ± 0.019	0.490 ± 0.118*	0.228 ± 0.127^#^	6.78 ± 0.30	8.53 ± 0.31*	6.92 ± 0.61^#^
4	0.186 ± 0.012	0.534 ± 0.127*	0.262 ± 0.190^#^	6.85 ± 0.29	9.65 ± 0.36*	8.48 ± 0.53^#^
6	0.201 ± 0.016	0.653 ± 0.227*	0.373 ± 0.125^#^	6.83 ± 0.25	12.69 ± 0.59*	9.56 ± 0.96^#^
8	0.221 ± 0.011	0.770 ± 0.114*	0.430 ± 0.224^#^	6.92 ± 0.21	15.35 ± 1.12*	10.15 ± 0.93^#^

*Note*. *Compared with Group A, *P* < 0.05; ^#^compared with Group B, *P* < 0.05.

**Table 2:** Comparison of W/D and PI.

Time (h)	PI			W/D		
	A	B	C	A	B	C
0	1.35 ± 0.41	1.35 ± 0.41	1.35 ± 0.41	4.267 ± 0.021	4.267 ± 0.021	4.267 ± 0.021
2	1.38 ± 0.54	2.15 ± 0.63*	1.56 ± 0.63^#^	4.253 ± 0.018	4.373 ± 0.025*	4.318 ± 0.022^#^
4	1.43 ± 0.36	2.89 ± 0.78*	1.78 ± 0.45^#^	4.298 ± 0.012	4.528 ± 0.030*	4.450 ± 0.020^#^
6	1.49 ± 0.25	3.95 ± 0.66*	1.95 ± 0.78^#^	4.405 ± 0.010	4.563 ± 0.215*	4.493 ± 0.025^#^
8	1.56 ± 0.32	4.87 ± 1.18*	2.12 ± 0.56^#^	4.490 ± 0.014	4.923 ± 0.224*	4.601 ± 0.018^#^

*Note*. *Compared with Group A, *P* < 0.05; ^#^compared with Group B, *P* < 0.05.

**Table 3:** TNF-*α*, IL-6 concentration in rat's serum and BALF (pg/mL, $\bar{x}\pm s$, *n* = 6).

Time (h)	Plasma			BALF		
	A	B	C	A	B	C
TNF-*α*						
0	133.9 ± 13.7	133.9 ± 13.7	133.9 ± 13.7	68.6 ± 11.7	68.6 ± 11.7	68.6 ± 11.7
2	203.5 ± 14.7	500.8 ± 23.6*	253.3 ± 20.7^#^	76.7 ± 31.6	130.17 ± 25.6*	86.5 ± 21.5^#^
4	300.6 ± 13.9	615.0 ± 21.0*	431.7 ± 22.3^#^	83.7 ± 21.7	173.33 ± 38.5*	103.5 ± 32.5^#^
6	367.1 ± 12.7	754.2 ± 59.3*	493.6 ± 50.5^#^	91.3 ± 27.8	207.17 ± 29.5*	136.8 ± 26.2^#^
8	416.8 ± 22.6	838.1 ± 30.6*	535.0 ± 28.3^#^	106.7 ± 23.4	258.33 ± 34.4*	176.2 ± 32.6^#^
IL-1*β*						
0	9.16 ± 1.47	9.16 ± 1.47	9.16 ± 1.47	5.17 ± 1.47	5.17 ± 1.47	5.17 ± 1.47
2	13.00 ± 1.96	28.17 ± 1.33*	10.2 ± 3.65^#^	7.83 ± 1.94	16.33 ± 3.68*	10.63 ± 2.39^#^
4	15.50 ± 1.84	47.17 ± 2.34*	16.7 ± 2.18^#^	9.67 ± 2.07	29.00 ± 3.92*	15.38 ± 3.64^#^
6	18.83 ± 1.75	71.83 ± 4.76*	26.2 ± 3.21^#^	10.33 ± 3.21	40.17 ± 4.38*	19.26 ± 5.21^#^
8	25.33 ± 2.33	92.16 ± 5.31*	36.2 ± 2.95^#^	12.00 ± 2.16	51.33 ± 6.16*	23.56 ± 2.91^#^
IL-6						
0	216.2 ± 12.5	216.2 ± 12.5	216.2 ± 12.5	112.6 ± 13.6	112.6 ± 13.6	112.6 ± 13.6
2	260.6 ± 11.6	417.5 ± 35.3*	296.5 ± 33.6^#^	128.3 ± 19.4	220.3 ± 37.8*	180.4 ± 23.6^#^
4	320.2 ± 20.4	617.3 ± 45.4*	356.7 ± 45.1^#^	166.7 ± 32.7	339.0 ± 32.2*	220.6 ± 25.6^#^
6	383.7 ± 27.2	703.5 ± 57.6*	433.5 ± 36.5^#^	193.3 ± 25.6	450.1 ± 50.8*	290.5 ± 36.8^#^
8	413.2 ± 32.5	816.9 ± 66.6*	523.6 ± 42.6^#^	260.0 ± 40.0	571.3 ± 76.6*	350.3 ± 24.8^#^
IL-10						
0	23.16 ± 3.64	23.16 ± 3.64	23.16 ± 3.64	10.91 ± 5.47	10.91 ± 5.47	10.91 ± 5.47
2	25.00 ± 4.26	58.17 ± 2.33*	50.98 ± 5.23	12.83 ± 1.94	21.33 ± 3.78*	19.32 ± 2.93
4	27.50 ± 3.04	67.17 ± 6.14*	65.73 ± 4.35	16.67 ± 3.07	29.00 ± 3.52*	26.37 ± 4.36
6	31.83 ± 4.72	71.83 ± 5.56*	69.32 ± 762	16.33 ± 5.61	26.17 ± 3.08*	23.12 ± 3.61
8	35.33 ± 5.73	62.16 ± 6.61*	60.42 ± 5.26	11.00 ± 4.00	21.33 ± 4.26*	19.12 ± 4.02

*Note*. *Compared with Group A, *P* < 0.05; ^#^compared with Group B, *P* < 0.05. Note: *compared with Group A, *P* < 0.05; ^#^compared with Group B, *P* < 0.05.

**Table 4:** The content of MDA, NO in the lung tissue ($\bar{x}\pm s$, *n* = 6).

Time (h)	MDA (nmol/mg)			NO (*μ*mol/mg)		
	A	B	C	A	B	C
0	2.86 ± 0.21	2.86 ± 0.21	2.86 ± 0.21	3.41 ± 0.24	3.41 ± 0.24	3.41 ± 0.24
2	2.91 ± 0.44	10.15 ± 0.53*	4.06 ± 0.33^#^	3.51 ± 0.19	9.53 ± 0.27*	4.82 ± 0.32^#^
4	2.99 ± 0.26	15.99 ± 0.76*	4.78 ± 0.37^#^	3.65 ± 0.15	14.65 ± 0.31*	5.06 ± 0.31^#^
6	3.09 ± 0.28	19.65 ± 0.58*	5.95 ± 0.68^#^	3.73 ± 0.21	16.69 ± 0.51*	5.95 ± 0.29^#^
8	3.26 ± 0.32	23.87 ± 0.98*	7.12 ± 0.56^#^	3.92 ± 0.28	18.35 ± 0.68*	6.24 ± 0.52^#^

*Note*. *Compared with Group A, *P* < 0.05; ^#^compared with Group B, *P* < 0.05.

## References

[1] Uchino S, Kellum JA, Bellomo R (2005). Acute renal failure in critically ill patients: a multinational, multicenter study. *JAMA*.

[2] Awad AS, Okusa MD (2007). Distant organ injury following acute kidney injury. *American Journal of Physiology*.

[3] Paladino JD, Hotchkiss JR, Rabb H (2009). Acute kidney injury and lung dysfunction: a paradigm for remote organ effects of kidney disease?. *Microvascular Research*.

[4] Doi K, Ishizu T, Fujita T, Noiri E (2011). Lung injury following acute kidney injury: kidney-lung crosstalk. *Clinical and Experimental Nephrology*.

[5] Singbartl K (2011). Renal-pulmonary crosstalk. *Contributions to Nephrology*.

[6] Scheel PJ, Liu M, Rabb H (2008). Uremic lung: new insights into a forgotten condition. *Kidney International*.

[7] Faubel S (2008). Pulmonary complications after acute kidney injury. *Advances in Chronic Kidney Disease*.

[8] Feltes CM, Van Eyk J, Rabb H (2008). Distant-organ changes after acute kidney injury. *Nephron*.

[9] Hassoun HT, Grigoryev DN, Lie ML (2007). Ischemic acute kidney injury induces a distant organ functional and genomic response distinguishable from bilateral nephrectomy. *American Journal of Physiology*.

[10] Feltes CM, Hassoun HT, Lie ML, Cheadle C, Rabb H (2011). Pulmonary endothelial cell activation during experimental acute kidney injury. *Shock*.

[11] Grigoryev DN, Liu M, Hassoun HT, Cheadle C, Barnes KC, Rabb H (2008). The local and systemic inflammatory transcriptome after acute kidney injury. *Journal of the American Society of Nephrology*.

[12] White LE, Hassoun HT (2012). Inflammatory mechanisms of organ crosstalk during ischemic acute kidney injury. *International Journal of Nephrology*.

[13] Klein CL, Hoke TS, Fang WF, Altmann CJ, Douglas IS, Faubel S (2008). Interleukin-6 mediates lung injury following ischemic acute kidney injury or bilateral nephrectomy. *Kidney International*.

[14] Hoke TS, Douglas IS, Klein CL (2007). Acute renal failure after bilateral nephrectomy is associated with cytokine-mediated pulmonary injury. *Journal of the American Society of Nephrology*.

[15] Borbiev T, Birukova A, Liu F (2004). p38 MAP kinase-dependent regulation of endothelial cell permeability. *American Journal of Physiology*.

[16] Ishii T, Doi K, Okamoto K (2010). Neutrophil elastase contributes to acute lung injury induced by bilateral nephrectomy. *American Journal of Pathology*.

[17] Kramer AA, Postler G, Salhab KF, Mendez C, Carey LC, Rabb H (1999). Renal ischemia/reperfusion leads to macrophage-mediated increase in pulmonary vascular permeability. *Kidney International*.

[18] Steinberg J, Halter J, Schiller H (2005). Chemically modified tetracycline prevents the development of septic shock and acute respiratory distress syndrome in a clinically applicable porcine model. *Shock*.

[19] Dos Remedios CG, Chhabra D, Kekic M (2003). Actin binding proteins: regulation of cytoskeletal microfilaments. *Physiological Reviews*.

[20] Dorion S, Landry J (2002). Activation of the mitogen-activated protein kinase pathways by heat shock. *Cell Stress and Chaperones*.

[21] Jiang Y, Gram H, Zhao M (1997). Characterization of the structure and function of the fourth member of p38 group mitogen-activated protein kinases, p38*δ*. *The Journal of Biological Chemistry*.

[22] Lavoie JN, Hickey E, Weber LA, Landry J (1993). Modulation of actin-microfilament dynamics and fluid phase pinocytosis by phosphorylation of heat shock protein 27. *The Journal of Biological Chemistry*.

[23] Mounier N, Arrigo A-P (2002). Actin cytoskeleton and small heat shock proteins: how do they interact?. *Cell Stress and Chaperones*.

[24] Gerthoffer WT, Gunst SJ (2001). Invited review: focal adhesion and small heat shock proteins in the regulation of actin remodeling and contractility in smooth muscle. *Journal of Applied Physiology*.

